# Effect of Pesticide Vinclozolin Toxicity Exposure on Cardiac Oxidative Stress and Myocardial Damage

**DOI:** 10.3390/toxics11060473

**Published:** 2023-05-23

**Authors:** Alessio Filippo Peritore, Gianluca Antonio Franco, Francesco Molinari, Alessia Arangia, Livia Interdonato, Ylenia Marino, Salvatore Cuzzocrea, Enrico Gugliandolo, Domenico Britti, Rosalia Crupi

**Affiliations:** 1 Department of Veterinary Science, University of Messina, 98168 Messina, Italy; aperitore@unime.it (A.F.P.); gianluca.franco@studenti.unime.it (G.A.F.); rcrupi@unime.it (R.C.); 2 Department of of Chemical, Biological, Pharmaceutical and Environmental Science, University of Messina, 98166 Messina, Italyrnglss92r55f251l@studenti.unime.it (A.A.); interdonatol@unime.it (L.I.);; 3 Department of Pharmacological and Physiological Science, School of Medicine, Saint Louis University, Saint Louis, MO 63104, USA; 4 Department of Health Sciences, “Magna Græcia University” of Catanzaro, Campus Universitario “Salvatore Venuta” Viale Europa, 88100 Catanzaro, Italy; britti@unicz.it

**Keywords:** pesticides, cardiotoxicity, oxidative stress

## Abstract

(1) Background: Vinclozolin is a popular fungicide used in fruit, ornamental plants, and vegetable crops. It has recently been seen that prolonged exposure to VZN can cause human or animal health damage to various organs, but little is known to date about its cardiovascular effects. In this study, we addressed the chronic effects of VZN on the myocardium and the enzymes involved in the cardiovascular function. (2) Methods: The animals were divided into four groups: group 1 served as the control, group 2 received 1 mg/kg of VZN by gavage, group 3 received 30 mg/kg of VZN by gavage, and group 4 received 100 mg/kg of VZN by gavage, for 30 days. (3) Results: Results showed that 100 mg/kg VZN markedly increased the plasma concentration of cardiac markers (CK-MB, cTnT, ANP, BNP). Moreover, compared to the control group, VZN treatment decreased the activity of SOD, CAT, and GPx, and downregulated the mRNA expression levels of Nrf2. Furthermore, collagen deposition was amplified owing to 100 mg/kg VZN cardiotoxicity. This harmful effect was confirmed by a histological study using hematoxylin and eosin (H&E) and Masson’s trichrome staining. (4) Conclusion: Overall, our results proved the cardiotoxicity caused by chronic exposure to VZN.

## 1. Introduction

In agriculture, the massive use of chemicals to increase productivity has resulted in the release of contaminants into the environment that can harm both human and animal health [1,2,3]. Vinclozolin [3-(3,5-dichlorophenyl)-5-methyl-5-vinyloxazolidine-2,4-dione] (VCZ) is a fungicide, widely used in Europe and the United States [4], for the control of *Sclerotinia sclerotiorum*, *Monilinia* spp., and *Botrytis cinerea* on vegetables, fruits, grapevine, and ornamental plants, as well as in the wine industry [5,6]. Several scientific studies have shown that vinclozolin used in agriculture can become a dangerous environmental and aquatic pollutant [7,8,9]. VCZ can be dispersed into the environment in several ways, hydrolyzed by microbes, abiotic degradation, or transported through water.

VCZ dispersion causes residues and its primary metabolites to accumulate in water bodies (levels range from 0.1 to 2.4 μg/L, up to a maximum detected level of 52 μg/L) [10,11,12,13,14]. Dietary and non-food exposure to VCZ and its metabolites has been analyzed by the U.S. Environmental Protection Agency (U.S. EPA). According to the studies conducted, it was determined that the various fungicides used in agricultural practice can circulate through both water and air, making contamination of treated and untreated food possible. However, it remains to be ascertained how much VCZ is directly ingested by animals or people through fruits and vegetables, as it depends greatly on the frequency and amount with which any contaminated food is consumed. VCZ is a known endocrine disruptor (EDC), which actions interfere not only with the immune system, but also with the biosynthesis, metabolism, and action of hormones. In fact, it was showed that VCZ can affect reproduction and fertility in both fish and mammals by decreasing sperm activity and sex organs development [10,13,14,15,16,17,18,19]. The toxicity effects from prolonged exposure to VCZ have been well documented. In fact, previous studies have shown how 28-day VCZ exposure can cause histological alteration and fibrosis in several organs such as lung and kidney on mouse [20,21]. The long-term damage caused by VCZ can be even greater, such as damage at behavioral level, affiliative behavior, or motor inhibition in mammals, but also in different class of animals like birds. In addition, renal damage from chronic VCZ exposure has also been seen in fish and in the liver metabolic profile of aquatic organisms [11,12,22,23,24]. Studies on the toxic action of VCZ have shown the exposure to this pesticide to be a risk factor in different time windows, not only in the long term. Exposure of pregnant rats to VZN has been shown to lead to a transgenerational epigenetic phenotype of disease in animals exposed during embryonic gonadal development [25,26,27]. Furthermore, VCZ exposure during testis differentiation alters the programming of germ cells and/or Sertoli cells, which cause germ cell apoptosis and reduced sperm motility later in adult life [28]. It has also been seen that VCZ exposure significantly affected the growth rate, gut microbial community composition, and functional pathways in an aquatic turtle, *P. sinensis* [29]. In a pharmacokinetics study, after oral administration of 100 mg/kg VCZ, it was found that levels of this pesticide were present in almost all organs analyzed, but not at the same concentration. In fact, a high level of VZC was observed in the first few hours in fat and liver, but little in serum and other tissues taken into analysis. Moreover, after 8 h, serum levels of the metabolite 3′,5′-dichloro-2,3,4-trihydroxy-2-methylbutylanilide (M5) were high [30]. Behind the mechanism of VCZ toxicity appears to be the involvement of oxidative stress, which leads to an imbalance in cellular homeostasis with increased production of markers of inflammation [31]. For example, in a study on testicular toxicity of VCZ following oral intake, the pesticide was linked to increased oxidative stress with decreased activity of antioxidant enzymes and increased lipid peroxidation in rats [20,21]. These results are in line with studies that relate the toxic action of environmental contaminants to the production of free radicals and increased oxidative stress [32]. In addition, many studies have shown how chronic exposure to contaminants can cause damage to the myocardium. Pathways leading to cardiovascular effects of particulate matter exposure have been mainly linked to oxidative stress, pulmonary, and systemic inflammation, endothelial cell dysfunction, atherosclerosis, and altered cardiac autonomic function [33]. Based on previous studies in which exposure to 100 mg/kg VCZ for 30 days caused multi-organ damage, we decided to investigate whether this concentration can also cause cardiac damage. In this study, we focused on the effects of VCZ at the cardiac level.

## 2. Materials and Methods

### 2.1. Animals

Male Sprague–Dawley rats (250 g, Envigo, Milan, Italy) were housed in a controlled cage (room 22.1 °C, 12 h dark-light cycles) and fed normal rodent food and water. For one week, the animals were acclimatized to these conditions. All animal studies followed the Italian legislation (D.Lgs 2014/26), as well as EU regulations (EU Directive 2010/63). The research was authorized by the University of Messina’s Animal Care Review Board (n°409\2022-PR).

### 2.2. Experimental Groups

Rats were randomly assigned to different groups, the doses and route of administration were based on previous studies [21]. The different concentrations of VCZ were dissolved in 0.45 mL of corn oil. Rats were gavaged as reported in the groups:

- Control (CTRL) n = 6: rats were orally administered the vehicle (corn oil) for 30 days.
- VCZ 1 mg/kg n = 6: rats were orally administered vinclozolin (1 mg/kg) for 30 days.
- VCZ 30 mg/kg n = 6: rats were orally administered vinclozolin (30 mg/kg) for 30 days.
- VCZ 100 mg/kg n = 6: rats were orally administered vinclozolin (100 mg/kg) for 30 days.

At the end of the experiment, animals were euthanized with an intraperitoneal overdose of ketamine and xylazine, and whole blood and heart tissues were collected from rats for further assays.

### 2.3. Heart Weight, hw/Body Weight, bw (hw/bw)

The rat’s body weight was measured daily during the experiment, from Day 0 to Day 30. At Day 30, the rats were sacrificed, and hearts were isolated to calculate relative hw/bw.

### 2.4. Histological Analysis

Heart tissues were fixed at room temperature in buffered formaldehyde solution (10% in PBS) (Sigma, St. Louis, MO, USA) for 24 h, dehydrated using a graded series of ethanol solutions (Sigma, St. Louis, MO, USA), embedded in Paraplast (Sherwood Medical, Mahwah, NJ, USA), and cut into 7-micrometer-thick sections with a pfm rotary 3004 microtome (Leica Microsystems SpA, Milan, Italy) [34]. Sections were deparaffinized with xylene (Bio-Optica, Milano, Italy) and stained with hematoxylin and eosin (Bio-Optica, Milano, Italy) [35], Furthermore, Masson’s trichrome staining was performed for the detection of collagen according to the manufacturer’s protocol (Bio-Optica, Milan, Italy), as reported previously [36]. Sections were evaluated using a Leica DM6 microscope (Leica Microsystems SpA, Milan, Italy) equipped with a motorized stage and associated with the Leica LAS X Navigator software (Leica Microsystems SpA, Milan, Italy) [37]. Histologic score was evaluated as described above [38]. Based on the score value, it is interpreted as:

- Negative score (score 0 or 1), the absence of myocardial damage.
- Positive score (score 2–7), the existence of damage
  - Mild myocardial damage (score 2–3),
  - Moderate myocardial damage (score 4–5),
  - Extensive myocardial damage (score 6–7).

### 2.5. Assessment of the Antioxidant System

The activity of the antioxidant enzymes superoxide dismutase, catalase, and glutathione peroxidase (SOD, CAT, and GPx) was carried out as previously described [39,40,41,42,43,44]. The protein concentration of the supernatant was estimated by Lowry method [45], using bovine serum albumin as a standard.

### 2.6. ELISA Measurement in Serum Samples

ELISA was performed as previously described [46,47]. Serum was obtained at day 30 of VCZ exposure. The levels of serum myocardial enzymes including creatine kinase isoenzyme (CK-MB) (CSB-E14403r), atrial natriuretic peptide (ANP) (CSB-E12982r), brain natriuretic peptide (BNP) (CSB-E07972r), and cardiac troponin T (cTnT) (CSB-E16443r) were determined using an enzyme-linked immunosorbent assay (ELISA) method (Cusabio), according to the protocol provided by the manufacturer.

### 2.7. Western Blot Analysis

Western blot analyses were done as previously described [48]. The following primary antibodies were used: anti-Bax (1:500, SCB, #sc7480), anti-Bcl-2 (1:500, SCB, sc-7382), and anti-caspase-3 (1:500, SCB, sc-56053), in 1 PBS, 5% *w*/*v* non-fat dried milk and 0.1% Tween-20 at 4 °C overnight. Blots were also incubated with primary antibody against GAPDH protein (1:5000, SCB, sc-32233), used as internal standard [49]. Signals were detected with an enhanced chemiluminescence detection system reagent according to the manufacturer’s instructions (SuperSignalWest Pico Chemiluminescent Substrate, Pierce) [50]. The relative expression of the protein bands was quantified by densitometry with Bio-Rad ChemiDocTMXRS+software (Biorad, Hercules, CA, USA) and standardized to β-actin levels. Images of blot signals (8-bit/600-dpi resolution) were imported into analysis software (Image Quant TL, v2003).

### 2.8. Terminal Deoxynucleotidyl Nick-End Labeling (TUNEL) Assay

Heart tissues were fixed at room temperature in a buffered formaldehyde solution (10% in PBS) for 24 h, dehydrated using a graded series of ethanol solutions, embedded in Paraplast (Sherwood Medical, Mahwah, NJ, USA), and cut into 7-mm thick sections. Sections were deparaffinized with xylene, and apoptosis was analyzed by a TUNEL assay using an in situ cell death detection kit (Roche, Basel, Switzerland, 11684795910) [51].

### 2.9. Real Time PCR

To evaluate the mRNA expression of target genes, RNA was extracted using an RNeasykit (Qiagen, Milan, Italy) for real-time polymerase chain reaction (PCR) analysis. RNA was quantified with a spectrophotometer (NanoDrop Lite; Thermo Fisher Scientific, Wilmington, DE, USA) [52,53]. An iScript RT-PCR kit (Bio-Rad, Hercules, CA, USA) was used to synthesize first-strand cDNA according to the manufacturer’s recommendations. In total, 1 μL [52,53] of total cDNA was used to perform real-time PCR analysis with the SYBRGreen method on a StepOnePlus Real-Time PCR System (Applied Biosystems, Waltham, MA, USA) [53]. GAPDH was used as an internal control for normalizing the relative expression levels between samples. For each target gene, besides the biological replicates, three technical replicates were performed. Negative controls using RNA as a template were also included in all runs to test for possible genomic DNA contamination of the samples.

### 2.10. Statistical Evaluation

For multiple comparisons, a two-way/one-way ANOVA was used, followed by a Bonferroni post-hoc test. Graphpad Prism 8 was used for statistical analysis. Statistically significant differences of the mean values are indicated by asterisks.

## 3. Results

### 3.1. Body Weigh, and Heart Weight and Histology

**Figure 1:**
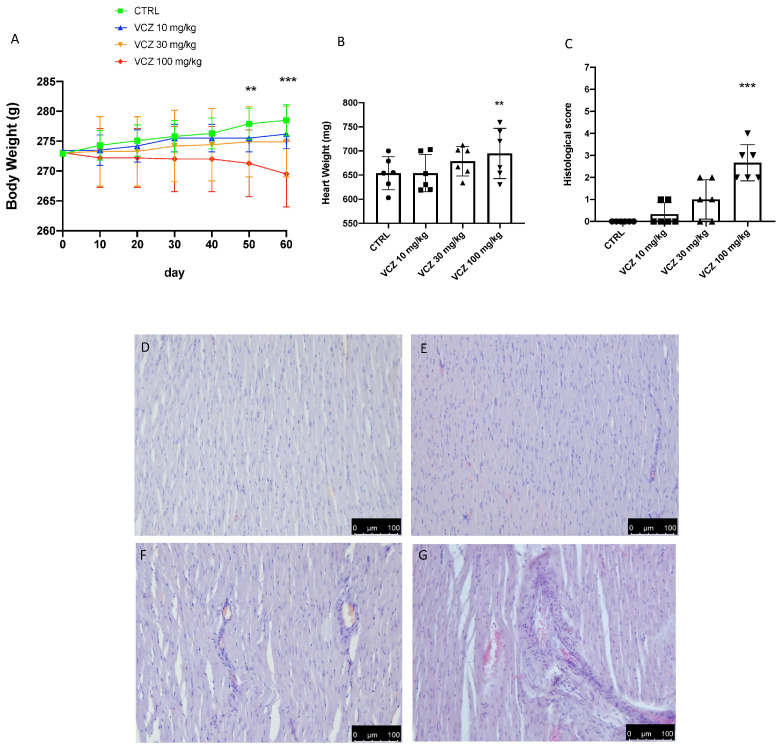
Effect of VCZ on body weight (gr) (**A**), heart weight (in mg) (**B**), and histological score (**C**) in normal CTRL and VCZ 1, 30, and 100 mg/kg exposed rats. Heart histology: H&E staining: magnification 40× of (**D**) CTRL; (**E**) VCZ 1 mg/kg; (**F**) VCZ 30 mg/kg; (**G**) VCZ 100 mg/kg. Values are expressed as mean ± SEM of six rats in each group. A *p*-value lower than 0.05 was considered significant: ** *p* < 0.01 vs. CTRL; *** *p* < 0.001 vs. CTRL.

Figure 1 shows the effect of VCZ after 30 days on body and heart weights in rats. At the end of experimental period, rats exposed to 100 mg/kg VCZ showed a significant decrease of their body weight compared to the CTRL group. Exposure to 1 and 30 mg/kg VCZ did not show difference in body weight after 30 days. Moreover, after 30 days of VCZ exposure, we observed an increase of the heart weight compared to the CTRL, but only in the VCZ 100 mg/kg group. No significant difference was observed in heart weight in both VCZ 1 and 10 mg/kg groups. Chronic exposure to VCZ caused a decrease in body weight in animals treated with 100 mg/kg, while no significant differences were observed for doses of 1 and 30 mg/kg (Figure 1A). A significant difference was detected in heart tissues (Figure 1). We decided to explore the histological damage in the interstitial and perivascular areas, after exposure to different concentrations of VCZ. Figure 1 presents a comparison of the microarchitecture of the heart among the experimental groups of the control and VCZ-treated animals. We observed that chronic exposure to 100 mg/kg of VCZ induced significant inflammatory infiltration in the heart. On the other hand, there were no signs of inflammation in VCZ 1 and 30 mg/kg groups, as well as in the CTRL group.

### 3.2. Heart Fibrosis

**Figure 2:**
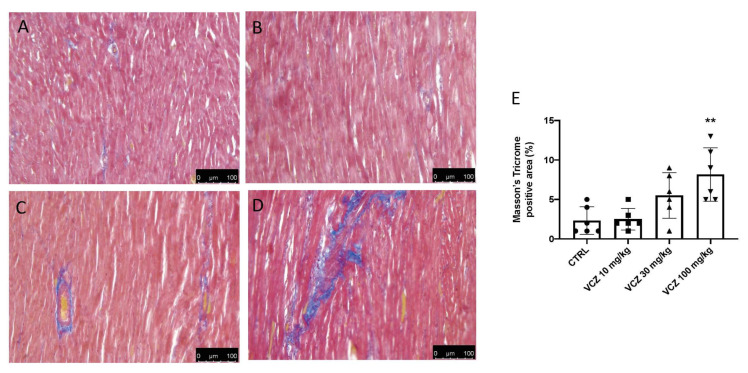
Collagen deposition after exposure to VCZ. Representative Masson’s trichrome staining of perivascular area at 30-day. (**A**) CTRL; (**B**) VCZ 1 mg/kg; (**C**) VCZ 30 mg/kg (**D**) VCZ 100 mg/kg; and (**E**) Masson’s Trichrome staining positive area %. A *p*-value lower than 0.05 was considered significant: ** *p* < 0.01 vs. CTRL.

Cardiotoxicity can lead to myocardial fibrosis with collagen accumulation in the heart. Masson’s trichrome staining showed increased collagen bundles, in blue, in rats exposed to 100 mg/kg VCZ, among myocardial sections commonly colored in red. In contrast, the increase in interstitial fibrosis concentration at 100 mg/kg was not found in the groups exposed to both 1 and 30 mg/kg VCZ compared with the control group (Figure 2).

### 3.3. Myocardial Enzymes Measurement in Serum

**Figure 3:**
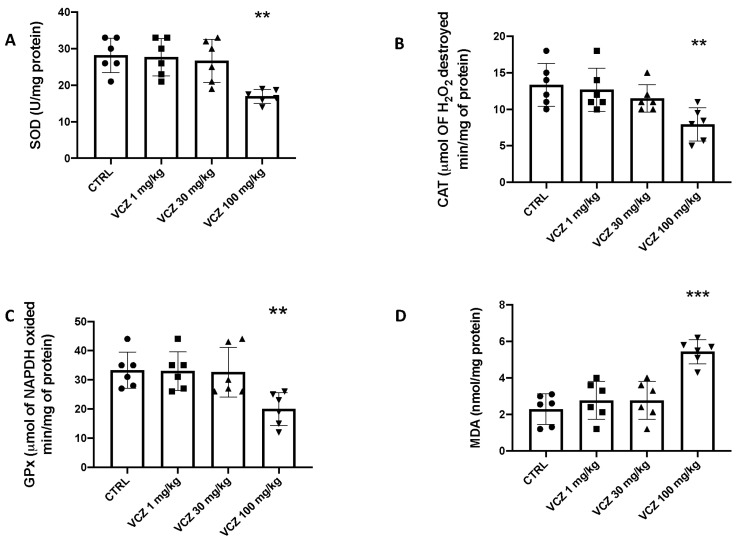
Serum levels of myocardial enzymes. (**A**) CK-MB, (**B**) cTnT, (**C**) ANP, (**D**) BNP. Values are expressed as mean ± SEM of six rats in each group. A *p*-value lower than 0.05 was considered significant. ** *p* < 0.01 vs. CTRL; *** *p* < 0.001 vs. CTRL.

Another parameter associated with cardiotoxicity is the analysis of serum levels of myocardial enzymes. The histological damage induced by exposure to 100 mg/kg VCZ is also reflected by the myocardial enzyme expression with increased levels of creatine kinase isoenzyme (CK-MB), atrial natriuretic peptide (ANP), brain natriuretic peptide (BNP), and cardiac troponin T (cTnT). In contrast, no changes were observed in the levels of the same myocardial enzymes following 1 and 30 mg/kg VCZ exposure (Figure 3).

### 3.4. Oxidative Homeostasis and Lipid Peroxidation

**Figure 4:**
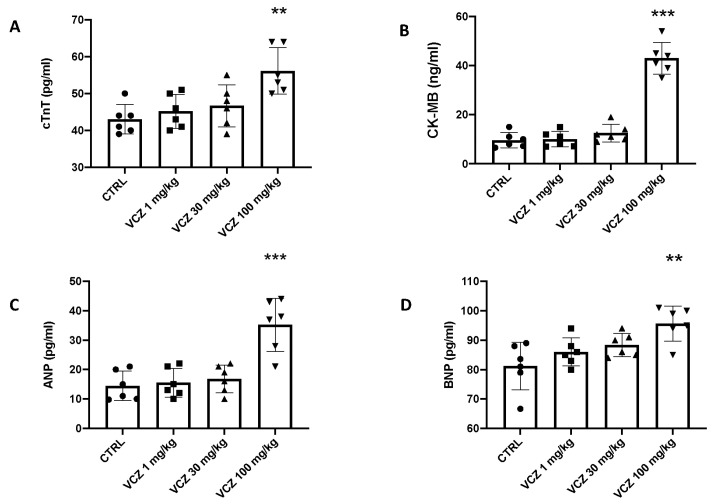
Values are expressed as mean ± SEM of six rats in each group. Effects of VCZ exposure on antioxidant response: (**A**) SOD activity; (**B**) CAT activity; (**C**) GPx activity, (**D**) MDA content. A *p*-value lower than 0.05 was considered significant. ** *p* < 0.01 vs. CTRL; *** *p* < 0.001 vs. CTRL.

The toxic action of VCZ is often related to an imbalance of antioxidant enzymes. As shown in Figure 4, exposure to 100 mg/kg VCZ showed a marked decrease in the activity of antioxidant enzymes (SOD, CAT, and GPx) in the heart tissue, but only in the high dose group compared to the control and low VCZ dose groups. In addition, the values of MDA, a marker of lipid peroxidation, were also observed. The amount of MDA in the hearts of rats treated with 100 mg/kg VCZ increased significantly compared with the control group. In contrast, no increase in MDA was detected following chronic exposures to 1 and 30 mg/kg VZN.

**Figure 5:**
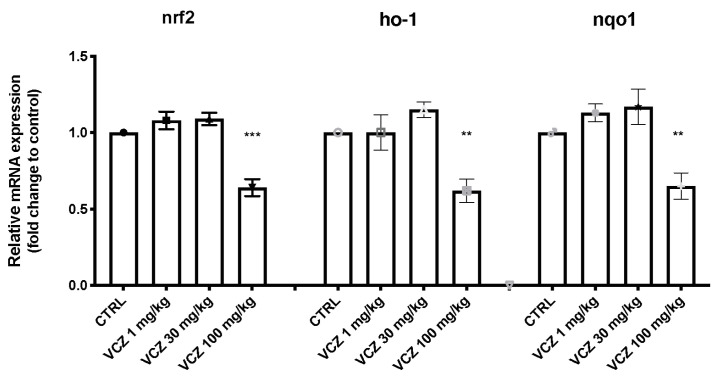
Effects of VCZ exposure on the oxidative stress response: Relative mRNA levels of (**A**) nuclear factor erythroid 2-related factor 2 (Nrf2); Heme oxygenase (HO-1) and NAD(P)H quinone dehydrogenase-1 (nqo1). A *p*-value lower than 0.05 was considered significant: ** *p* < 0.01 vs. CTRL; *** *p* < 0.001 vs. CTRL.

The nuclear factor erythroid 2-related factor 2 (Nrf2) pathway has a key role in the amelioration of oxidative injury. Real-time analysis showed the downregulation of Nrf2 mRNA on samples harvested from the VCZ 100 mg/kg group compared to CTRL. Moreover, our results showed that the chronic 100 mg/kg VCZ exposure downregulated the expression of some genes downstream Nrf2, heme oxygenase (HO-1) and NAD(P)H quinone dehydrogenase-1 (NQO-1), contrary to the other two VCZ groups (1 and 30 mg/kg), compared to CTRL (Figure 5).

### 3.5. Apoptotic Pathway

**Figure 6:**
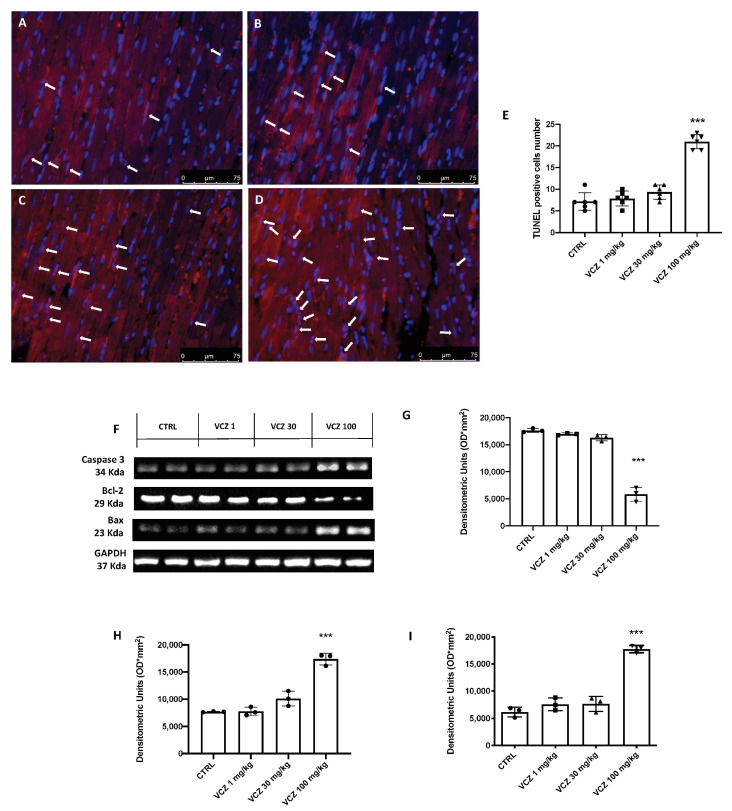
TUNEL assays indicated an abnormal apoptotic pattern. TUNEL positive apoptotic cells (white arrow) in rats exposed to VCZ at different doses. CTRL (**A**), VCZ 1 mg/kg (**B**), VCZ 30 mg/kg (**C**), VCZ 100 mg/kg (**D**). Effects of VCZ on protein levels of apoptotic pathway (caspase-3, Bax and Bcl-2). Western blot analysis (**E**). Each figure corresponds to a representative replicate from three experiments: blots (**F**) and the corresponding histogram for densitometric analysis, for caspase-3, Bax and Bcl-2 (**G**–**I**). Values = means ± SD of three independent experiment data: *** at *p* < 0.001 against CTRL.

TUNEL assays were performed to study VCZ-induced apoptosis. After 30 days of chronic VCZ exposure at a dose of 100 mg/kg, an increase of apoptotic cells was observed compared with the control group, while the groups with VCZ exposure at 1 and 30 mg/kg showed no significant increases. The data on the apoptotic process were confirmed by western blot analysis of pro (caspase-3 and Bax) and anti-apoptotic (Bcl-2) proteins. Following chronic exposure to VCZ at a dose of 100 mg/kg, it was observed a significant increase in the levels of the pro-apoptotic protein caspase-3, as well as Bax, with a concomitant decrease in the expression of anti-apoptotic protein Bcl-2. Non-significant changes were observed for the groups exposed to VCZ at doses of 1 and 30 mg/kg compared with the control group (Figure 6).

## 4. Discussion

The increased accumulation of chemicals in the environment, especially anthropogenic chemicals, poses a huge risk to animals and humans. Environmental factors can be a source of oxidative stress for animal species, which occurs when the amount of reactive oxygen species produced exceeds the counteracting capacity of antioxidants [40,41]. Most of the studies on VCZ focus mainly on the reproductive system. The VZN toxicity is often associated with increased oxidative stress, for example when ingested in excessive amounts and repeatedly in fish models [20,21]. Previous studies have shown a link between pesticide exposure and cardiotoxicity. In fact, it has been shown that pesticide exposure can cause side effects on myocardial tissues, such as altered myocardial contractility leading to heart failure, myocardial fiber degeneration, and connective tissue edema [54,55,56]. Although the toxic action of prolonged VCZ exposure on various organs has been extensively studied, little is known about the potential cardiotoxic effects. Previous studies have shown how 100 mg/kg VCZ can cause damage in several organs with continuous exposure for 28 days [20,21]. Also in the same study, it was seen that a lower dose, 30 mg/kg VCZ, did not show significant alterations to lung tissues, probably due to an insufficiently long exposure interval [20]. It was seen that sublethal doses of contaminants, like pesticides, for prolonged exposure, can cause cardiotoxicity [57]. Therefore, we focused on the potential cardiotoxic effects induced by VCZ chronic exposure at different lethal and nonlethal doses.

In the present study, there was a significant decrease in weight body weight and an increase in cardiac weight following chronic exposure to high-dose VCZ. The increase in cardiac weight could be due to increased water content or edematous intramuscular space, while the loss of body weight could be due to reduced food intake related to the animal’s sickness. Histopathology analysis on heart sections of animals exposed and not exposed to the different concentrations of VCZ confirmed the hypotheses. In fact, H&E staining on heart sections showed increased damage with vacuolization of cardiomyocytes, infiltration of inflammatory cells, separation of myocardial tissue, and loss of myofibrils, in a VCZ concentration-dependent manner. Following the loss of cardiomyocytes, an increase in fibrotic tissue known as reparative fibrosis develops. Reparative fibrosis is stimulated by cardiomyocyte necrosis and is essential to counteract volume loss. Cardiac fibrosis that develops in response to the loss of cardiomyocytes is considered reparative fibrosis, an essential reparative response to injury and cell death. Unlike reparative fibrosis, reactive fibrosis is the term used for the mechanism which occurs in the absence of cell death with a diffuse collagen deposition throughout the myocardium. It occurs in the absence of cell death and can be stimulated by prolonged periods of stress or by exposure to profibrotic mediators. While reparative fibrosis plays an important role in maintaining the integrity of the heart after myocardial infarction, reactive fibrosis is clearly a less beneficial process. Myocardial interstitial edema was observed in heart samples from animals exposed to VCZ, as well as increased collagen deposition leading to fibrotic tissue formation. These observations are in accordance with previous studies [58,59]. Myocardial enzymes (CK-MB, cTnT, ANP, BNP) are biochemical cardiac injury markers which are novel tools to evaluate the cardiac injury because of high sensitivity and specificity [60,61]. The serum levels of CK-MB, ANP, BNP are elevated in various heart diseases such as hypertrophy, heart failure, MI, and so on [62]. Results showed that the serum levels of CK-MB, ANP, BNP, and cTnT after exposure to high VCZ doses were significantly higher than the low doses treated group or the control. Previously, it has been widely demonstrated that prolonged exposure to pesticides can cause an increase in reactive oxygen species (ROS) resulting in increased oxidative stress and decreases in the activity of antioxidant systems [63,64,65]. Overproduction of ROS can cause oxidative damage to nucleic acids, but also to lipids, carbohydrate proteins, and, ultimately, cellular, and structural injury. The body uses several defense mechanisms against ROS accumulation and increased oxidative stress, known as the antioxidant mechanism. SOD catalyzes the dismutation of the superoxide radicals O_2_ to H_2_O_2_, while CAT reduces the H_2_O_2_ into H_2_O and O_2_, which is the most effective way of protecting cell and maintaining cell homeostasis [66]. Glutathione-related enzymes such as glutathione peroxidase (GPx) act directly or indirectly as antioxidants. Malondialdehyde is known as the main product of peroxidized polyunsaturated fatty acids and MDA levels have been used extensively as an important indicator of lipid peroxidation [67]. In the present study, our results showed a significant increase in MDA in the hearts of rats exposed to different doses of VZN. The increase in cardiac lipid peroxidation levels following VCZ exposure in a concentration-dependent manner is in line with what has been observed in previous studies on rats exposed to the same pesticide [20,21]. The increase in lipid peroxidation clearly indicates oxidative stress due to VCZ exposure, and this also led to a decrease in antioxidant defenses in cardiac tissue in the long term. We can hypothesize that ROS affect antioxidant defense mechanisms by limiting the enzymatic activity of SOD, CAT, and GPx, resulting in increased levels of MDA, as demonstrated previously. In fact, lipid peroxidation clearly shows that oxidative stress has been established in treated rats, which leads to a depletion in enzymatic (SOD, CAT, and GPx) activity in the heart tissue. Additionally, in a system where oxidant species predominate, oxidative stress is defined as an imbalance between oxidant and antioxidant species [68].

One of the most important survival and cellular defense pathways to challenge toxicants and oxidative stress is the Nrf2 signalling pathway [69]. Under unstressed conditions, the cytoplasmic inhibitor Keap1 keeps Nrf2 in a quiescent state. Under oxidative conditions, the cytoplasmic complex is wrecked and Nrf2 translocates into the nucleus to induce the transcription of phase II detoxifying and antioxidant enzymes [70]. Our data showed that VCZ exposure deactivated the Nrf2 signalling pathway, weakening the activity of antioxidant enzymes, including CAT, SOD, and GPx.

As a cell suicide mechanism, apoptosis can get rid of superfluous or undesired cells [71]. The caspase family has a critical function in cell apoptosis, and caspase-3 has been shown to be an important executor that is activated downstream in apoptosis pathways [72]. Previous studies have demonstrated the ability of VCZ to activate the intrinsic apoptotic pathway, which is normally regulated by caspase-3-dependent activation [27]. In our study, the exposure to high doses of VCN, but not the low doses or the control, lead to induction of apoptosis, accompanied by a significant increase in caspase-3 expression, in line with what has been observed previously in other organs [20,21]. The increase in oxidative stress caused the activation of caspase-3 and Bax, while reducing the expression of Bcl-2 [73]. In the present study, we demonstrated how the fungicide Vinclozolin induced chronic cardiotoxicity after 30 days of exposure, with increased expression of the apoptosis-inducing target genes and reduced expression of the antiapoptotic factor. However, several researchers have shown that commercially used pesticides in formulations with other substances, e.g., surfactant, have higher toxicity of single active ingredients. Many studies have revealed that pesticides, such as glyphosate end-use products (formulations), are more toxic to aquatic organisms than the glyphosate acid active ingredient alone, and that this depends on the toxicity of the POEA, a surfactant used in the formulation [74,75,76,77]. In our study, we focused on the toxicity of a single active ingredient, the pesticide Vinclozolin, and gave an overview of its effects on the heart. More studies will be needed on the toxicity synergies of the various substances in the formulations and their degree of hazard to the species that come into contact with them.

## 5. Conclusions

Prolonged and repeated exposure to VCN can induce cardiac injury and thus cardiotoxicity. Indeed, VCZ ingested over long periods of time can cause tissue changes and fibrosis in a concentration-dependent manner, followed by increased levels of biomarker enzymes of cardiac injury. Specifically, the VCZ concentration of 100 mg/kg reduced antioxidant capacity and increased lipid peroxidation in exposed rats. VCZ exposure induced increased collagen deposits in the myocardial tissue, accompanied by increased myocardial enzymes and apoptosis.

## Data Availability

Not applicable.
